# Humans do not perceive conspecifics with a greater exposed sclera as more trustworthy: a preliminary cross-ethnic study of the function of the overexposed human sclera

**DOI:** 10.1007/s10211-018-0296-5

**Published:** 2018-08-22

**Authors:** Dariusz P. Danel, Sławomir Wacewicz, Zdzisław Lewandowski, Przemysław Żywiczyński, Juan Olvido Perea-Garcia

**Affiliations:** 1 Polish Academy of Sciences, Unit of Anthropology, Hirszfeld Institute of Immunology and Experimental Therapy, ul. Rudolfa Weigla 12, 53-144 Wroclaw, Poland; 2 Department of Heart Diseases, Wroclaw Medical University, Wroclaw, Poland; 3 Center for Language Evolution Studies, Faculty of Languages, Nicolaus Copernicus University, Torun, Poland; 4 Department of Human Biology, Faculty of Physiotherapy, University School of Physical Education in Wroclaw, Wroclaw, Poland; 5 Interacting Minds Centre, Aarhus University, Aarhus C, Denmark

**Keywords:** Human eye, Trustworthiness, White sclera, Exposed sclera size index SSI, Cooperative eye hypothesis

## Abstract

Understanding the adaptive function of the unique morphology of the human eye, in particular its overexposed white sclera, may have profound implications for the fields of evolutionary behavioural science, and specifically the areas of human interaction and social cognition. Existing hypotheses, such as the cooperative eye hypothesis, have attracted a lot of attention but remain untested. Here, we: (i) analysed variation in the visible sclera size in humans from different ethnic backgrounds and (ii) examined whether intraspecific variation of exposed sclera size is related to trust. We used 596 facial photographs of men and women, assessed for perceived trustworthiness, from four different self-declared racial backgrounds. The size of the exposed sclera was measured as the ratio between the width of the exposed eyeball and the diameter of the iris (sclera size index, SSI). The SSI did not differ in the four examined races and was sexually monomorphic except for Whites, where males had a larger SSI than females. In general, the association between the SSI and trustworthiness was statistically insignificant. An inverted U-shaped link was found only in White women, yet the strength of the effect of interaction between sex and race was very small. Our results did not provide evidence for the link between exposed sclera size and trustworthiness. We conclude that further investigation is necessary in order to properly assess the hypotheses relating to the socially relevant functions of overexposed sclera.

For us humans, the eyes are not only sensory organs but, when observed by others, also an extremely important socio-cognitive stimulus. The wealth of information conveyed by the human eyes includes cues of health and age (Russell et al. 2014; Gründl et al. 2012), social interest (Kret et al. 2014), emotional and attentional states (Provine et al. 2013; Hess and Polt 1960) as well as behavioural intent (Adams and Kleck 2005) and male dominance (Kleisner et al. 2010; but see also: Kocnar et al. 2012, who did not confrim the association between eye morphology and male dominance). This informative function is underscored by the uniqueness of the ocular morphology in our species (Kobayashi and Kohshima 1997). In their original study, Kobayashi and Kohshima (1997) compared the ocular morphology of 188 primate species and concluded that humans present a uniquely conspicuous morphology that enhances the perception of gaze by others. It has further been hypothesised that the development of the remarkably overexposed white sclera was an adaptive response to a new cooperative niche in the socio-ecological environment of our hominin ancestors (the cooperative eye hypothesis; Tomasello et al. 2007).

Although the underlying relation between human eye morphology and cooperative behaviour assumes evolutionary origins, the exact nature of this link remains unspecified. A common interpretation is that Tomasello et al. (2007) focus on the perceptual affordances of our conspicuous eye, which facilitates establishing common ground and result in a more efficient alignment of intentions in collaborative tasks (cf. e.g. Perea García et al. 2017). Crucially, when viewed from the perspective of evolutionary behavioural science, the emergence and stability of cooperative behaviour are subject to strong game-theoretic constraints (e.g. Axelrod and Hamilton 1981; cf. e.g. Wacewicz et al. 2017). On such accounts, *trust* is often treated as the critical element of cooperation (Balliet and Van Lange 2013; Boone and Buck 2003; Ferrin et al. 2008), with the operationalisations of “trust” and “cooperation” being to some extent interchangeable (cf. a trust game, e.g. Chen et al. 2012). Interesting in the context of our own research is that this approach is taken in recent studies of pupil size and synchrony in pupillary changes, which have attested to a relationship between human ocular morphology and cooperation in the trust game paradigm (Kret and De Dreu 2017; Kret et al. 2015).

In this work, we empirically test the value of the human eye as a marker of trustworthines. Specifically, we examine—in a multi-ethnic design—whether intraspecific variation of sclera size in men and women is related to their perceived trustworthiness. We predict that a more exposed sclera (as reflected by higher values of the sclera size index—or SSI; detailed definition: see the “Methods” section) is related to a higher perceived trustworthiness.

The present study is the first empirical approach to testing the perceptual effects of variation of sclera size in humans. Therefore, the secondary aim of our study is to provide a systematic analysis of SSI variation in men and women from different racial backgrounds.

## Methods

### Stimuli

We acquired our stimulus from the Chicago Face Database (CFD; Ma et al. 2015). The CFD is a publicly available database of facial photographs categorised into “races”^1^ and with extensive norming data, including objective measures (dimensions of facial traits, luminance, etc.) as well as subjective ratings (age, attractiveness, dominance, trustworthiness, etc.). Trustworthiness was asessed there on a 1–7 scale (1—not at all; 7 extremely); assessments were elicited by the following instruction: “Now, consider the person pictured above and rate him/her with respect to other people of the same race and gender. (For example, if you indicated that the person was Asian and male, consider this person on the following traits [here: trustworthiness] relative to other Asian males in the United States)” and yielded a very high reliability (α = .99; more details: Ma et al. (2015). Subjective ratings for the original CFD were done by a convenience sample of 1087 raters, out of which 552 were female (and 227 did not report their sex). The raters self-reported diverse racial backgrounds, but the majority were White (516 White, 117 Asian, 74 Black, 72 biracial or multiracial, 57 Latino, 18 other and 233 did not report their racial background). The CFD version 2.0.3 used in the current study is an extended version of the database described in Ma et al. (2015) and includes updated norming data and a greater number of *White* and *Black* faces relative to the earlier version, as well as facial photographs of *Asians* and *Latinos*. We used stimuli from all of these “races”. For many faces in the CFD, several emotional facial expressions are available, but for our analyses, we used only faces with a “neutral” facial expression.

### Measurements

We used the exposed sclera size index (SSI; Kobayashi and Kohshima 1997)—the width of the exposed eyeball (the distance between the corners of the eye excluding *caruncula lacrimalis*) divided by the diameter of the iris—as our measure of the size of the area of the visible sclera. The reason behind this choice is that this is an established measure in the literature. For each of the photographs, the SSI was calculated as a mean of the SSI values for both eyes of the photographed face. A total of 596^2^ faces (*n* = 196 *Black*, *n* = 103 female; *n* = 183 *White*, *n* = 90 female; *n* = 109 *Asian*, *n* = 57 female; *n* = 108 *Latino*, *n* = 56 female) were measured by one person (ZL) on an LCD computer monitor with Adobe Photoshop Creative Suite version 5. We used the ruler tool scaled to 1 pixel = 1.0000 pixels (menu analysis > set measurements scale > pixel length = 1; logical length = 1; logical units = pixels).

### Statistical analyses

In the first exploratory step of the statistical analysis, we examined the possible effects of sex and race on the SSI by conducting a two-way ANOVA. In the second step of the analysis, we examined the association between perceived trustworthiness and the SSI allowing for the sex and race of an individual. Initial visual inspection of scatterplots for perceived trustworthiness, and the SSI suggested that in some components of the model (groups defined by sex and race), the association between both variables is curvilinear (inverted u-shape). In order to formally test linear and quadratic effects of continuous predictors at different levels of categorical variables, we employed general linear modelling (GLM) for separate slopes with both linear and quadratic terms for the SSI. Thus, in our final analytical model, perceived trustworthiness was a dependent variable, sex and race were categorical predictors, and the SSI and SSI-squared were continuous predictors.

## Results

**Fig. 1:**
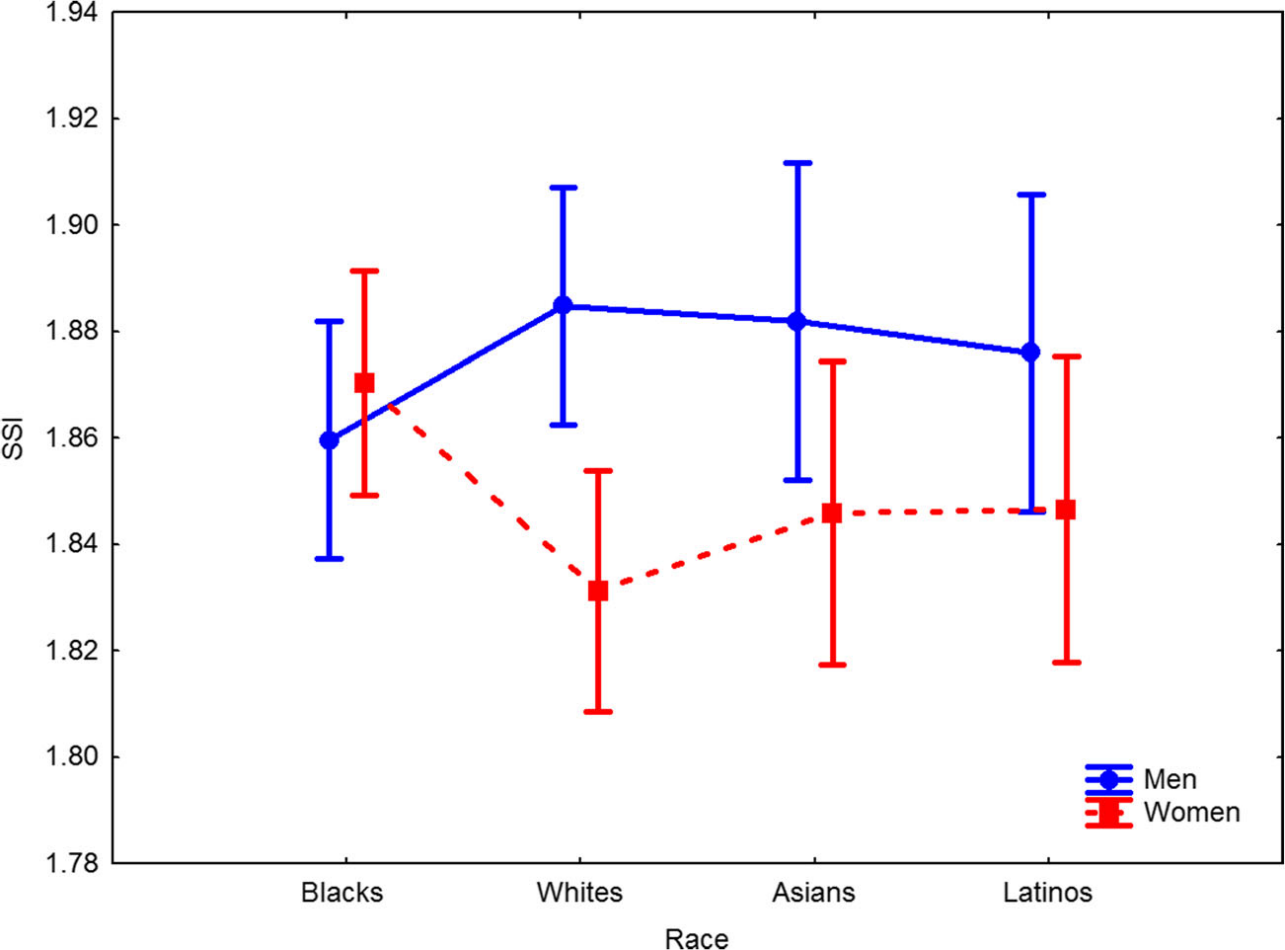
Sclera size index (SSI) by individuals’ race and sex. Vertical bars denote .95 confidence intervals

The two-way ANOVA showed that the sizes of the exposed sclera, as measured by the SSI, did not differ in the four examined races (*F*(3, 588) = .14, *p* = .94, *η*_*p*_^*2*^ = .001). A comparison of SSI values between sexes showed males to have larger sclera sizes than females (males: *M* = 1.87, females: *M* = 1.85; *F*(1, 588) = 8.42, *p* = .004, *η*_*p*_^*2*^ = .01). However, a detailed examination of statistically significant interaction between participants’ sex and race (*F*(3, 588) = 2.89, *p* = .035, *η*_*p*_^*2*^ = .01; Fig. 1) with post-hoc Tukey’s test showed that the difference in sclera size between males and females was significant for White faces (males: *M* = 1.88, 95% CI [1.86, 1.91], females: *M* = 1.83, 95% CI [1.81, 1.85], *p* = .02), while non-significant for Asian (males: *M* = 1.88, 95% CI [1.85, 1.91], females: *M* = 1.85, 95% CI [1.82, 1.87], *p* = .70), Latino (males: *M* = 1.88, 95% CI [1.85, 1.91], females: *M* = 1.85, 95% CI [1.82, 1.88], *p* = .87) and Black faces (males: *M* = 1.86, 95% CI [1.84, 1.88], females: *M* = 1.87, 95% CI [1.85, 1.89], *p* = .99).

In the second step, the GLM model was statistically significant (*F*(23, 572) = 5.42, *p* < .0001) and explained *R*^*2*^ = 18% of variance in perceived trustworthiness ratings of the photographed individuals. In the model, neither race (*F*(3, 572) = 1.61, *p* = .19, *η*_*p*_^*2*^ = .008) nor sex (*F*(1, 572) = 2.71, *p* = .10, *η*_*p*_^*2*^ = .005) nor interaction between race and sex (*F*(3, 572) = .73, *p* = .54, *η*_*p*_^*2*^ = .004) significantly affected assessments of trustworthiness, implying that indeed, due to the specific instruction for trustworthiness ratings, participants assessed faces within the respective  sex and race categories.

**Fig. 2:**
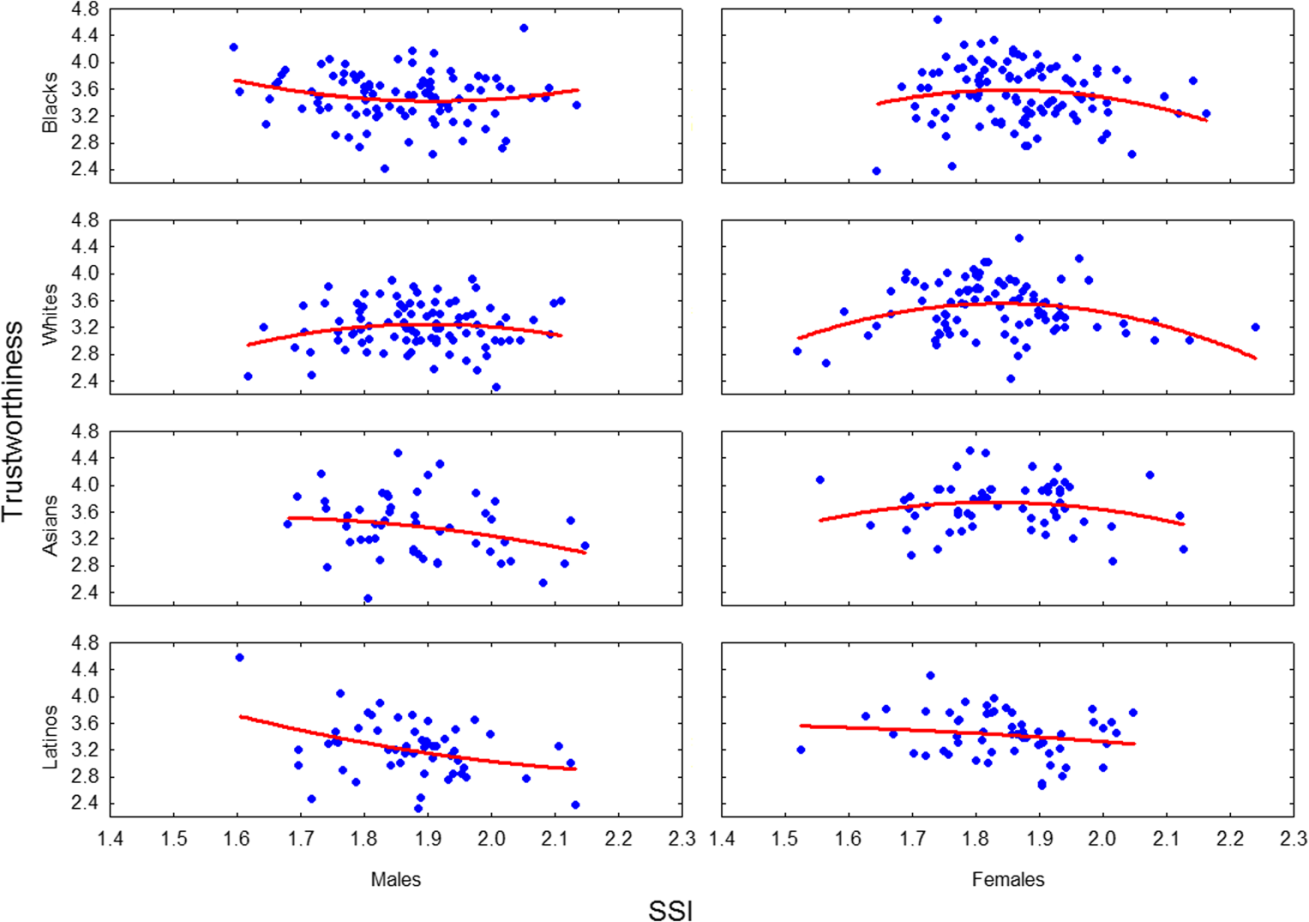
The association between perceived trustworthiness and exposed sclera size (SSI—sclera size index) categorised by sex and self-declared race. Note that the association was statistically significant only for White women (see details in the main text)

Confirming our predictions from the analysis of scatterplots, both the three-way interaction between race, sex and the SSI (*F*(8, 572) = 2.32, *p* = .02, *η*_*p*_^*2*^ = .03) as well as the interaction between race, sex and the SSI-squared (*F*(8, 572) = 2.35, *p* = .02, *η*_*p*_^*2*^ = .03) were statistically significant. Further analyses showed, however, that the association between perceived trustworthiness and the exposed sclera size was significant only for White women. Statistically significant regression coefficients for linear (*β* = 29.96, *t*(572) = 3.09, *p* = .002) and quadratic term (*β* = − 15.08, *t*(572) = − 3.13, *p* = .002) indicated that while in general, an increase in White women’s sclera size is associated with higher perceived trustworthiness, the relationship is not linear. In fact, the negative regression coefficient for the quadratic term indicated an inverted U-shaped relationship and a decrease in perceived trustworthiness for White women with large sclera sizes. Regression coefficients for the SSI and perceived trustworthiness in all the other racial and sex groups analysed in the model did not reach significance levels (for all |*t*(572)| < 1.77, *p* > .08). A summary of the results is presented in Fig. 2.

In the last step of the analysis, we formally tested whether adding the quadratic term to the analytical model was justified. The *F*-test comparing goodness of fit of GLM models with and without the SSI-squared term showed that the difference between the amount of variance explained by the two models (18% vs. 15%, respectively) was statistically significant (*F*(8, 572) = 2.35, *p* = .02). Thus, the model with the quadratic term fitted the data better.

## Discussion

Understanding the selective pressures that gave rise to the morphological particularities of the human eye has profound and far-reaching implications for understanding human evolution at large, including the origins of human species-specific sociality and communication (e.g. Tomasello et al. 2007). The cooperative eye hypothesis (Tomasello et al. 2007) proposes that a white sclera, and the enhanced gaze following that it affords, was selectively favoured in cooperative contexts. In the current study, we focused on trust, an important prerequisite for cooperation in humans.

Overall, despite measuring sclera size in almost 600 individuals from four racial backgrounds and using trustworthiness ratings from over 1000 judges, contrary to our hypothesis, a more exposed sclera (higher SSI) was not associated with a higher perceived trustworthiness. This association was only statistically significant for White women in the first two quartiles of the SSI distribution; however, the strength of this effect was very small. Our descriptive analyses showed no significant difference between the SSI of the four self-declared “races”, although within White faces, the SSI in males was significantly larger than in females.

Our results do not confirm the idea that humans are biased to perceive conspecifics with a greater exposed sclera size as more trustworthy partners in cooperative tasks. However, without discussing other factors that may have affected our results, we cannot unambiguously disconfirm a link between the human ocular morphology and judgements of cooperative dispositions.

The cross-ethnic character of our study might have been one of such factors since the attribution of characteristics such as trustworthiness is very sensitive to the context in which ratings are gathered. In the Chicago Face Database, used in the current study, the self-reported ethnic background of the raters was mixed, so many of the raters were exposed to faces of outgroups. It has been shown that racial identity may influence the activity of the brain regions responsible for emotional reactions (i.e. amygdala, which was also shown to activate during assessing trustworthiness of faces; Engell et al. 2007; Winston et al. 2002) when exposed to facial stimuli characteristic of outgroups (Hart et al. 2000; Phelps et al. 2000). As the neurophysiological correlates of amygdala activation include negative emotions such as fear and aggression, it cannot be ruled out that trustworthiness ratings in the CFD were influenced by the ethnic mismatch of the raters and stimuli. If this was the case, the association between trustworthiness and subtle morphological facial features such as sclera size might have been outweighed by a relatively strong effect of racial membership. Although in the current study the potential outgroup effect on trustworthiness judgements was at least partially controlled by the relativised procedure of the ratings (see the “Methods” and “Results” sections confirming the effectiveness of the applied methodology), our study should be replicated in a racially homogeneous design.^3^ Another complication that may have affected our results is related to the SSI itself. Although the SSI as the measure of exposed sclera is firmly established in the literature (Kobayashi and Kohshima 1997, 2001), it was developed for interspecific rather than intraspecific comparisons. Therefore, a variation of the SSI within the species could be too small for a reliable identification of psychological correlates of the human sclera. In fact, our results may have accounted for this effect—the SSI values were similar across the four “races”, and statistically significant sex differences were found only in individuals who declared themselves as White. Interestingly, only in White women, the SSI was associated with trustworthiness ratings.

The sexual dimorphism of the SSI found in Whites and the statistically significant association between the SSI and trustworthiness in White women are also intriguing in the context of the evolution of eye colour variation characteristic of Caucasian populations (Frost 2006, 2014). It has been proposed that diverse eye colouration observed in early Europeans and their descendants is a result of sexual selection that took place in the specific ecological conditions of northern and eastern Europe. Restrictions on polygyny and male shortage in this world region intensified sexual selection of women and evolution of novel morphological qualities, such as iridal colouration (Frost 2006, 2014). Whether similar sexual selection pressures affected the evolution of other ocular features, such as sclera size, and what the ultimate causes of the smaller sclera size were in White women remain to be tested in further research. Similarly, the shape of the link between SSI and trustworthiness in White women requires additional investigation. Although our results imply that in this group neither small (i.e. feminine) nor large (i.e. masculine) sclera are perceived trustworthy, the very low strength of the observed effect (*η*_*p*_^*2*^ = .03) makes thorough interpretation difficult.

The interactions with other facial features may also affect the association between ocular morphology and trustworthiness. Although the analysis of the influence of eye colour on trustworthiness was beyond the scope of the current study, previous research has shown that iris colouration may affect the perception of trustworthiness (Kleisner et al. 2013). Importantly, face shape was found to be a key factor influencing the correlation between eye colouration and trustworthiness ratings (Kleisner et al. 2013). Whether facial morphology, iris colouration or other ocular traits such as the limbal ring (Ilicic et al. 2016) directly or through the interactions affect the association between trustworthiness and sclera characteristics remains to be determined.

In conclusion, the present study is the first empirical test of the hypothesised link between exposed sclera size and perceived trustworthiness, a prerequisite for successful cooperation in humans. Although we did not find evidence supporting this hypothesis, we conclude that it is too early to dismiss the possibility that such a link exists. This research problem requires and deserves further studies, which will take into account the factors discussed above.

## Footnotes

1 The anthropological notion of “race” has well-known problems (e.g. Wagner et al. 2017); here, however, “race” is a socio-cultural category, with racial membership self-declared by the photographed person.

2 We excluded an individual coded as BF-203 because it was self-declared as black, while clearly representing a different phenotype.

3 CFD within-race trustworthiness ratings were not available as of 2017 (DS Ma, personal communication, November 03, 2017).
